# Ergebnisse eines monozentrischen Gefäßscreeningprogramms in Deutschland

**DOI:** 10.1007/s00104-023-01821-0

**Published:** 2023-02-20

**Authors:** K. Passek, U. Ronellenfitsch, K. Meisenbacher, A. Peters, D. Böckler

**Affiliations:** 1 Klinik für Gefäßchirurgie und Endovaskuläre Chirurgie, Universitätsklinikum Heidelberg, Im Neuenheimer Feld 420, 69120 Heidelberg, Deutschland; 2 Klinik für viszerale, Gefäß- und endokrine Chirurgie, Universitätsklinikum Halle (Saale), Ernst-Grube-Straße 40, 06120, Halle, Deutschland

**Keywords:** Screening, Karotisstenosen, Abdominelles Aortenaneurysma, Periphere arterielle Verschlusskrankheit, Risikofaktoren, Screening, Carotid stenosis, Abdominal aortic aneurysm, Peripheral arterial occlusive disease, Risk factors

## Abstract

**Hintergrund:**

Kardiovaskuläre Erkrankungen sind die häufigste Todesursache in Europa mit relevanter sozioökonomischer Belastung. Ein Screeningprogramm für Gefäßerkrankungen bei asymptomatischen Personen mit definierter Risikokonstellation kann zu einer frühen Diagnose führen.

**Ziel der Arbeit:**

Die Studie untersucht ein Screeningprogramm auf Karotisstenosen, periphere arterielle Verschlusskrankheit (pAVK) und abdominelle Aortenaneurysmen (AAA) bei Menschen ohne anamnestisch bekannte arterielle Gefäßerkrankungen hinsichtlich demografischer Daten, Risikofaktoren, Vorerkrankungen, Medikamenteneinnahme sowie Detektion und Therapie pathologischer Befunde.

**Material und Methoden:**

Probanden wurden durch verschiedene Informationsmaterialien eingeladen, ein Fragebogen erfasste kardiovaskuläre Risikofaktoren. Das Screening erfolgte mit ABI-Messung und Duplexsonographie als monozentrische, prospektive, einarmige Studie innerhalb eines Jahres. Endpunkte waren die Prävalenz von Risikofaktoren, pathologische und/oder behandlungsbedürftige Befunde.

**Ergebnisse:**

Es nahmen 391 Personen teil, bei 36,0 % bestand mindestens ein kardiovaskulärer Risikofaktor, 35,5 % wiesen zwei und 14,4 % drei oder mehr auf. Aus der Sonographie der Karotiden resultierte bei 9 % ein kontrollbedürftiger Befund mit einer < 50%igen bis > 75%igen Stenose oder eines Verschlusses. Ein AAA mit einem Durchmesser von 3,0–4,5 cm wurde bei 0,9 % nachgewiesen, ein pathologischer ABI < 0,9 oder > 1,3 bei 12,3 %. Bei 17,0 % bestand befundbasiert die Indikation einer Pharmakotherapie, bei keinem die einer Operation.

**Diskussion:**

Es zeigte sich die Durchführbarkeit eines Screeningprogramms auf das Vorliegen einer Karotisstenose, einer pAVK und eines AAA bei definierten Risikopersonen. Es wurden kaum behandlungsbedürftige Gefäßpathologien im Einzugsgebiet der Klinik nachgewiesen, sodass ein Einsatz des Programms in Deutschland in dieser Form derzeit auf Basis der Daten nicht empfohlen werden kann.

## Abstract (en)

**Background:**

Cardiovascular diseases are the main cause of death in Europe with a relevant socioeconomic burden. A screening program for vascular diseases in asymptomatic persons with a defined risk constellation can lead to an early diagnosis.

**Objective:**

The study examined a screening program for carotid stenosis, peripheral arterial occlusive disease (PAOD) and abdominal aortic aneurysms (AAA) in persons without any known vascular disease with respect to demographic data, risk factors, pre-existing conditions, medication intake, detection of pathological findings and/or findings requiring treatment.

**Material and methods:**

Test subjects were invited using various information material and filled in a questionnaire on cardiovascular risk factors. The screening took place with measurement of the ABI and duplex sonography as a monocentric prospective single arm study within 1 year. Endpoints were the prevalence of risk factors and pathological and/or results requiring treatment.

**Results:**

A total of 391 persons participated, 36% presented with at least 1 cardiovascular risk factor, 35.5% with 2 and 14.4% with 3 or more. The sonography showed results requiring control with a carotid stenosis of < 50–> 75% or occlusion in 9%. An AAA with a diameter of 3.0–4.5 cm was diagnosed in 0.9% and a pathological ABI < 0.9 or > 1.3 in 12.3%. The indications for a pharmacotherapy were found in 17% and no operation was recommended.

**Conclusion:**

The practicability of a screening program for carotid stenosis, PAOD and AAA of a defined risk population was shown. Vascular pathologies that required treatment were hardly found in the catchment area of the hospital. Consequently, the implementation of this screening program in Germany based on the collected data cannot currently be recommended in this form.

## Hintergrund und Fragestellung

Kardiovaskuläre Erkrankungen stellen in Europa die häufigste Todesursache mit etwa 4 Mio. Todesfällen (43 % aller Todesfälle) im Jahr 2016 dar [5, 10, 24, 25]. Im Jahr 2019 starben in Deutschland etwa 44.000 Menschen an einem Myokardinfarkt, dem Endstadium der koronaren Herzkrankheit [19]. Zusätzlich zur Mortalität sind kardiovaskuläre Erkrankungen aufgrund der hohen Behandlungskosten sowie der Morbidität mit Funktionseinschränkungen ein relevantes sozioökonomisches Problem. Jährlich erleiden mehr als 200.000 Menschen in Deutschland einen Apoplex, ursächlich hierfür kann eine Stenose der A. carotis interna sein. Auch zeigte sich in den letzten Jahrzehnten eine stetig relevante Zunahme der Prävalenz der peripheren arteriellen Verschlusskrankheit (pAVK), wobei von einer progredienten Entwicklung auszugehen ist [17]. In der 2001 in Deutschland durchgeführten Querschnittstudie „get ABI“ fanden sich bei nahezu 20 % der Teilnehmer nach Vollendung des 65. Lebensjahres eine pAVK [12]. Klinische Manifestationen der pAVK sind belastungsabhängige Schmerzen der unteren Extremität sowie trophische Störungen und Ulzerationen mit einer Gefährdung der Extremität und des Lebens durch mögliche septische Komplikationen. Eine weitere potenziell lebensbedrohliche kardiovaskuläre Erkrankung ist das abdominelle Aortenaneurysma (AAA). Seine Prävalenz wird in Registerstudien mit 4–8 % bei über 65-jährigen Männern und 0,5–1,5 % bei gleichaltrigen Frauen angegeben [9]. Das vom Maximaldurchmesser abhängige Rupturrisiko beträgt 3 % pro Jahr bei einem Durchmesser von 5 cm und 6 % pro Jahr bei einem Durchmesser von mehr als 7 cm [23]. Eine Ruptur ist trotz Fortschritten in den Behandlungsmethoden nach wie vor mit einer Gesamtletalität außer- und innerhalb des Krankenhauses von deutlich über 50 % behaftet.

Ähnliche Risikofaktoren begünstigen das Auftreten der beschriebenen kardiovaskulären Erkrankungen. Diese verlaufen in frühen Stadien meist asymptomatisch und werden oft erst spät diagnostiziert. Eine frühe Diagnosestellung ist essenziell, da diese zu deutlich besseren Ergebnissen führt als eine spätere oder eine Behandlung in einer Notfallsituation [5, 26]. Beispielhaft sind hier die Karotisendarteriektomie bei asymptomatischer Stenose zur Apoplexprophylaxe oder die elektive Ausschaltung eines AAA zur Rupturprophylaxe ebenso wie die pharmakologische Therapie von Risikofaktoren der Arteriosklerose zur Sekundärprophylaxe genannt [13, 18].

Aus dem geschilderten epidemiologischen Hintergrund ergibt sich die Annahme, dass ein Screening auf Gefäßerkrankungen bei asymptomatischen Personen, die eine definierte Risikokonstellation aufweisen, sinnvoll sein könnte. Gemäß Beschluss des Gemeinsamen Bundesausschlusses 2016 und Gesundheitsuntersuchungsrichtlinie 2020 haben gesetzlich krankenversicherte Männer ab 65 Jahren Anspruch auf eine einmalige Ultraschalluntersuchung der Bauchaorta hinsichtlich eines Aneurysmas [6, 7]. Ein Screening für andere Bevölkerungsgruppen wie z. B. weibliche Personen sowie ein Screening auf Stenosen der A. carotis oder eine pAVK sind nicht vorgesehen und somit für Versicherte in der gesetzlichen Krankenversicherung nicht zugänglich. Von privaten Krankenversicherungen werden Kosten dafür in der Regel nicht übernommen. Ein solches Screening könnte nichtinvasiv mittels Doppler‑/Duplexsonographie für die Karotisstenose sowie durch Bestimmung des Knöchel-Arm-Indexes (ABI) für die pAVK erfolgen. Beide Verfahren liefern wie auch das Ultraschallscreening auf das Vorliegen eines AAA in der Hand des erfahrenen Untersuchers schnell valide Aussagen über das Vorliegen der Erkrankungen Karotisstenose bzw. pAVK [14, 22]. Herausforderungen für ein solches Programm sind die Definition einer Population mit relevantem Risiko für ein Vorliegen der genannten Erkrankungen und es muss gewährleistet sein, dass möglichst viele Personen in dieser Population an der Untersuchung teilnehmen. In den USA wurde Anfang der 2000er-Jahre das Programm Dare to C.A.R.E, das ein Screening auf Karotisstenosen, AAA, pAVK und Nierenarterienstenosen einschloss, durchgeführt. Es zeigte sich hier ein relevanter Anteil behandlungsbedürftiger Gefäßerkrankungen [21].

Die vorliegende Studie soll ein Screeningprogramm auf das Vorliegen von Karotisstenosen, einer pAVK und eines AAA bei Menschen ohne bekannte arterielle Gefäßerkrankungen hinsichtlich Teilnahme, Detektion behandlungsbedürftiger Befunde und möglicher Weiterbehandlung evaluieren.

## Studiendesign und Untersuchungsmethoden

Das Screeningprogramm wurde in einer monozentrischen, prospektiven, einarmigen Studie explorativ evaluiert.

### Einschluss- und Zielkriterien

Die Einschluss- und Zielkriterien finden sich in Tab. 1.

**Table 1:** 

*Primäres Zielkriterium*	Prävalenz mindestens einer relevanten Gefäßerkrankung: Stenose der A. carotis interna ≥ 60 % gemäß NASCET-Kriterien [16] oder pAVK mit ABI ≤ 0,9 oder Erweiterung der abdominellen Aorta auf einen maximalen Transversaldurchmesser von ≥ 3 cm
*Sekundäre Zielkriterien*	Alter
	Geschlecht
	Grad einer Karotisstenose gemäß NASCET-Kriterien
	ABI
	Maximaler Transversaldurchmesser der abdominellen Aorta
	Prävalenz kardiovaskulärer Risikofaktoren
	Häufigkeit von Eingriffen infolge der Screeningbefunde: Start einer pharmakologischen Therapie, Operation bzw. endovaskuläre Therapie der Karotisstenose, des AAA und/oder aufgrund einer pAVK, kardiologische Intervention (z. B. Koronarangiographie)
*Einschlusskriterien*	Einwilligung in die Studienteilnahme nach ausführlicher Aufklärung bei gegebener Aufklärungsfähigkeit
	Keine bekannte arterielle Gefäßerkrankung (Karotisstenose, AAA, pAVK)
	Kein gefäßspezifisches Screening innerhalb von 5 Jahren vor Studienteilnahme
	Alter ≥ 60 Jahre oder Alter ≥ 50 Jahre und Vorliegen mindestens eines der folgenden anamnestischen Risikofaktoren: Hyperlipidämie, arterielle Hypertonie, aktiver Nikotinabusus, positive Familienanamnese für kardiovaskuläre Erkrankungen oder Alter ≥ 40 Jahre und Vorliegen eines Diabetes mellitus

*NASCET* „North American Symptomatic Carotid Endarterectomy Trial“, *pAVK* periphere arterielle Verschlusskrankheit, *ABI* Ancle-Brachia-Index, *AAA* abdominelles Aortenaneurysma

### Screeningprogramm

**Figure 1:**
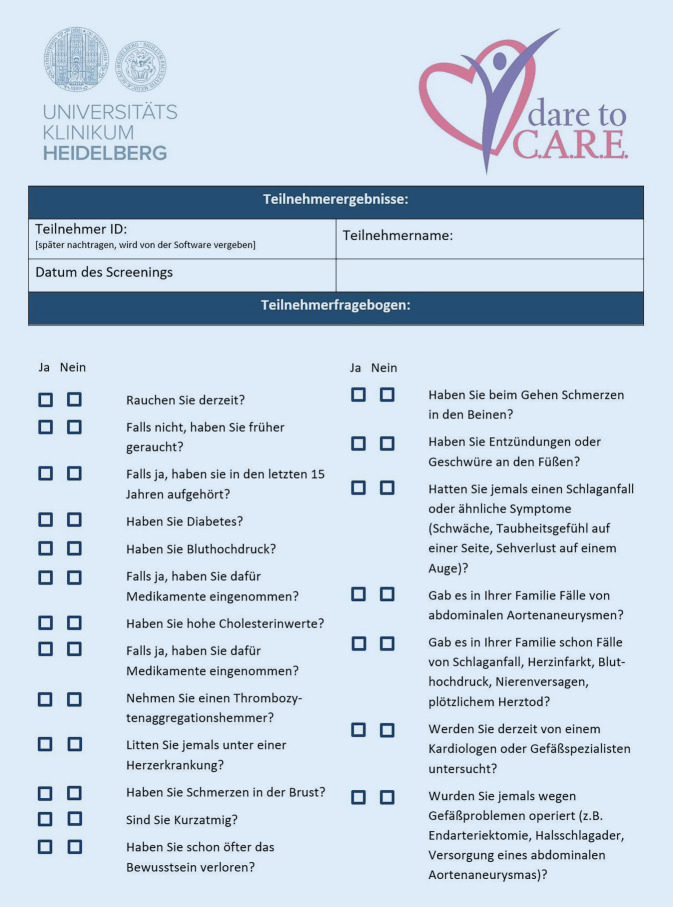

Das Screeningprogramm stellt eine Adaptation des ursprünglich in den USA etablierten Dare-to‑C.A.R.E.-Programms dar [21]. Von jedem Studienteilnehmer wurden mithilfe eines strukturierten Fragebogens (Abb. 1) relevante anamnestische Vorbefunde sowie demografische Daten erhoben, dann erfolgte das aus drei Komponenten bestehende Screening. Durch eine standardisierte B‑Bild-Sonographie sowie Doppler- und farbkodierte Duplexsonographie wurden beide Aa. carotides mit einem Linearschallkopf mit 5–7 MHz von DEGUM-Stufe I bzw. DEGUM-Stufe II (Deutsche Gesellschaft für Ultraschall in der Medizin) zertifizierten Fachärzten untersucht und der Grad einer eventuell vorliegenden Stenose gemäß der NASCET(„North American Symptomatic Carotid Endarterectomy Trial“)-Kriterien quantifiziert [2]. Die abdominelle Aorta wurde ebenfalls mittels B‑Bild-Sonographie mit einem Konvexschallkopf mit 2–8 MHz beurteilt und ihr maximaler Transversaldurchmesser bestimmt. Die ABI-Bestimmung erfolgte durch Blutdruckmessung und Tasten von Pulsen bzw. Ableitung eines CW-Dopplersignals mittels Stiftsonde an beiden oberen und unteren Extremitäten.

Die erhobenen Befunde wurden sowohl direkt mit dem Studienteilnehmer besprochen als auch durch einen Bericht, der weitere Behandlungsempfehlungen einschloss, an den Haus- bzw. Facharzt übermittelt. Eventuell erforderliche weitere diagnostische oder therapeutische Maßnahmen wurden durch den behandelnden Arzt in Absprache mit dem Studienteilnehmer geplant. Die Durchführung solcher Maßnahmen war nicht unmittelbar Gegenstand der Studie. Falls sich bei den Screeninguntersuchungen dringend behandlungsbedürftige Befunde ergaben, so wurde neben einer dezidierten Aufklärung des Studienteilnehmers telefonisch Kontakt mit dem primär behandelnden Arzt aufgenommen und ggf. auch eine sofortige stationäre Weiterbehandlung veranlasst.

### Rekrutierung und Aufklärung

Die Rekrutierung der Studienteilnehmer gelang primär über allgemein- und fachärztliche Praxen. Durch Poster und Informationsbroschüren für das Studienprojekt wurden die Praxen über die Möglichkeit der Studienteilnahme ihrer Patienten informiert. Zusätzlich wurden potenzielle Studienteilnehmer durch öffentliche Informationsveranstaltungen, Pressemitteilungen und soziale Medien zur Teilnahme eingeladen. Auch innerhalb des Universitätsklinikums Heidelberg wurden Informationsbroschüren für Patienten, Angehörige, Besucher und Mitarbeiter ausgelegt. Die ausführliche Aufklärung über Ziele und Ablauf der Studie erfolgte zum Zeitpunkt der Vorstellung im Studienzentrum vor Durchführung der studienspezifischen Maßnahmen.

### Statistische Verfahren

Aufgrund des explorativen Studiencharakters wurden die Zielgrößen rein deskriptiv ausgewertet. Für normal verteilte Zielkriterien wurden Mittelwert, Median sowie Standardabweichung und 95 %-Konfidenzintervalle bestimmt. Für nicht normalverteilte kontinuierliche Zielkriterien wurden Median, Minimum, Maximum sowie Interquartilsabstand, für diskret verteilte Zielkriterien Häufigkeiten mit 95 %-Konfidenzintervallen ermittelt. Die Prävalenzen von Erkrankungen und Risikofaktoren bzw. Häufigkeiten von Eingriffen wurden errechnet durch die Anzahl der Studienteilnehmer mit dem entsprechenden Merkmal dividiert durch die Gesamtzahl dieser. Da es sich um eine explorative Studie handelte, wurde auf eine spezifische Hypothese mit einhergehender Fallzahlberechnung verzichtet. Der Untersuchungszeitraum wurde auf ein Jahr limitiert.

### Rechtliche und ethische Aspekte

Die Untersuchung wurde in Übereinstimmung mit der Deklaration von Helsinki in ihrer aktuellen Fassung durchgeführt. Vor Studienbeginn wurde ein zustimmendes Votum der Ethikkommission der Medizinischen Fakultät Heidelberg eingeholt (S-719/2017).

## Ergebnisse

### Studienteilnehmer

Neben den allgemeinen Informationsmaßnahmen in den Medien sowie innerhalb des Universitätsklinikums Heidelberg wurden 1256 niedergelassene Fachärzte (417 Allgemeinmediziner, 84 Urologen, 755 Internisten) angeschrieben. An diese wurden 15.000 Flyer zur Auslage in den Praxen und Weitergabe an Patienten versandt. Im Studienzeitraum vereinbarten 439 Personen einen Termin in der Studiensprechstunde. Von diesen wurden 391 Personen (89,1 %) in die Studie eingeschlossen. 48 Personen (10,9 %) erschienen entweder nicht zum vereinbarten Termin oder erfüllten die Einschlusskriterien nicht. Der Hauptanteil der Studienteilnehmer folgte der Einladung über eine direkte Empfehlung bzw. den Erhalt von Informationsmaterial beim Haus- oder niedergelassenen Facharzt, wobei der jeweilige Rekrutierungsweg in Tab. 2 zusammengefasst ist.

**Table 2:** 

Zuweisungs- bzw. Rekrutierungsweg	Anzahl	Anteil (%)
Haus-/niedergelassener Facharzt	183/391	46,8
Mundpropaganda	94/391	24,0
Spezifische Infomedien (Presse)	20/391	5,1
Sonstige (Krankenhausmitarbeiter, Internet, andere)	28/391	7,1
Keine Angabe	66/391	16,9

Die demografischen Merkmale der Studienteilnehmer sowie die jeweiligen Einschlusskriterien sind in Tab. 3 aufgeführt.

**Table 3:** 

*Geschlecht*	**Anzahl (Anteil)**
Weiblich	227/391 (58,1 %)
Männlich	164/391 (41,9 %)
*Alter*	
Mittelwert	65,1 Jahre
Spannweite	40–84 Jahre
*Einschlusskriterien*	
40–49 Jahre, Diabetes mellitus	4/391 (1,0 %)
50–59 Jahre, ein kardiovaskulärer Risikofaktor	110/391 (28,1 %)
≥ 60 Jahre	272/391 (69,5 %)
Einschlusskriterien formal nicht erfüllt	5/391 (1,3 %)

Bei 36,0 % der Studienteilnehmer bestand eigenanamnestisch mindestens ein kardiovaskulärer Risikofaktor, 35,5 % wiesen zwei und 14,4 % drei oder mehr Risikofaktoren auf (Tab. 4).

**Table 4:** 

Anzahl kardiovaskulärer Risikofaktoren	Anzahl (Anteil)
0	55 (14,1 %)
1	140 (36,0 %)
2	138 (35,5 %)
3	47 (12,1 %)
4	9 (2,3 %)

Die Häufigkeiten der in den Screeninguntersuchungen erhobenen Befunde zeigt Tab. 5. Für alle Untersuchungen fällt ein geringer Anteil weiter abklärungsbedürftiger Befunde auf. Aus der Sonographie der Karotiden resultierte bei 9 % der Teilnehmer ein kontrollbedürftiger Befund, bei keinem Teilnehmer eine Pathologie, bei der eine starke Empfehlung zu einer operativen oder interventionellen Therapie bestand. Bei 2 Teilnehmern zeigte sich mit einer Karotisstenose von 60–75 % ein Befund, bei dem eine operative oder interventionelle Behandlung in Erwägung zu ziehen war. Bei etwa 1 % der Teilnehmer fand sich ein AAA, für das Kontrollen empfohlen werden mussten. Ein AAA mit einem Durchmesser, bei dem gemäß der gültigen deutschen S3-Leitlinie eine operative oder endovaskuläre Behandlung empfohlen wurde, lag bei keinem Teilnehmer vor. Für den ABI wurde bei etwa 12,3 % der Teilnehmer eine Pathologie erhoben, wobei zu fast gleichen Teilen falsch hohe Indizes als Zeichen einer Mediasklerose und eines möglichen Diabetes mellitus als auch erniedrigte Indizes als Zeichen einer pAVK auftraten. Bei keinem Teilnehmer wurde ein Eingriff unmittelbar basierend auf den Screeningbefunden empfohlen. Bei 2 Studienteilnehmern mit einer Karotisstenose von 60–75 % Stenosegrad wurde angeraten, eine Intervention bzw. Operation in Erwägung zu ziehen. Bei 67 Studienteilnehmern (17,0 %) war der Beginn einer Pharmakotherapie indiziert.

**Table 5:** 

Befund	Häufigkeit
*Arteria carotis interna rechts/links*	
Keine Stenose	705/782 (90,2 %)
Stenose < 50 % (Kontrolle empfohlen)	61/782 (7,8 %)
Stenose 50–60 % (Kontrolle empfohlen)	5/782 (0,6 %)
Stenose 60–75 % (Intervention zu erwägen)	2/782 (0,3 %)
Stenose > 75 % (Intervention empfohlen)	0/782 (0 %)
Verschluss (angiologische Abklärung empfohlen)	2/782 (0,3 %)
Kein Befund erhoben	7/782 (0,9 %)
*Maximaler Transversaldurchmesser der Aorta abdominalis*	
< 3 cm (kein Aneurysma)	387/391 (99,0 %)
3,0–3,9 cm (Kontrolle innerhalb 24 Monaten)	3/391 (0,8 %)
4,0–4,5 cm (Kontrolle innerhalb 12 Monaten)	1/391 (0,3 %)
> 4,5 cm (Kontrolle innerhalb 6 Monaten)	0/391 (0 %)
> 5 cm (Intervention zu erwägen)	0/391 (0 %)
*ABI*	
Beide Werte 0,9–1,3 (nicht pathologisch)	111/391 (85,2 %)
Mindestens ein Wert 0,75–0,89 (leicht erniedrigt, angiologische Abklärung empfohlen)	23/391 (5,9 %)
Mindestens ein Wert 0,5–0,74 (deutlich erniedrigt, angiologische Abklärung empfohlen)	5/391 (1,3 %)
Mindestens ein Wert < 0,5 (extrem erniedrigt, angiologische Abklärung empfohlen)	0/391 (0 %)
> 1,3 (falsch hoch, Verdacht auf Mediasklerose, Abklärung hinsichtlich Diabetes mellitus empfohlen)	20/391 (5,1 %)
Kein Befund erhoben	10/391 (2,6 %)

*ABI* Ancle-Brachial-Index

## Diskussion

Die vorliegende Studie demonstriert die Durchführbarkeit eines Screenings auf Gefäßerkrankungen in Deutschland mit einer auf die Screeningkapazität bezogenen hohen Nachfrage in einer asymptomatischen Population, die anhand spezifischer Risikokriterien definiert wird. Eine genaue Responserate ließ sich nicht feststellen, da aufgrund der spezifischen Informationsmaßnahmen über niedergelassene Ärzte sowie Auslagen im Klinikum und persönliche Kontakte die exakte Anzahl der Personen, die eine Einladung zur Studienteilnahme erhalten hatten, nicht zu ermitteln war. Die geplante Anzahl an Teilnehmern wurde nahezu vollständig erreicht, wobei diese eher durch die Sprechstundenkapazitäten und die Laufzeit der Untersuchung als durch die Nachfrage limitiert wurde. Im Vergleich zum initialen Dare-to‑C.A.R.E.-Screeningprogramm in den USA, welches 2007 im ersten Jahr mit 125 Probanden begann, wurde hier eine deutlich höhere Anzahl erreicht [21]. Größere Teilnehmerzahlen wären ähnlich wie bei anderen Erhebungen bei Verlängerung des Untersuchungszeitraumes zu erwarten [5]. Da die Studie einen explorativen Charakter hatte, wurde keine Fallzahl a priori definiert. Basierend auf der in die Auswertungen einbezogenen Anzahl an Studienteilnehmern lassen sich nichtsdestotrotz Aussagen hinsichtlich Teilnahmebereitschaft, Profil der Teilnehmer sowie primärer und sekundärer Zielgrößen der Studie treffen.

Inwiefern die Population der Studienteilnehmer repräsentativ für die Bevölkerung der Untersuchungsregion ist, kann mit den vorliegenden Daten nicht beantwortet werden, da keine Informationen zur Grundgesamtheit der eingeladenen Personen bzw. zur Grundgesamtheit der Bevölkerung in der Untersuchungsregion vorliegen. Durch die Einladung potenzieller Studienteilnehmer über Haus- und Facharztpraxen sollten möglichst zielgerichtet Menschen erreicht werden, die die Einschlusskriterien für das Screeningprogramm hinsichtlich Alter und kardiovaskulärer Risikofaktoren erfüllen. In der überwiegenden Mehrzahl der Fälle stellen Teilnehmer an Screeningprogrammen jedoch eine spezifische Selektion der eingeladenen Population dar, da die Bereitschaft zur Teilnahme an Screeningprogrammen regelhaft mit demografischen und sozioökonomischen Charakteristika sowie dem Risikoprofil der einzelnen Screeningteilnehmer assoziiert ist [1, 8].

Sowohl Einladungen über Arztpraxen als auch Mundpropaganda waren für die Rekrutierung maßgeblich. Die vorgegebenen Einschlusskriterien prägten die Charakteristika der Studienteilnehmer, Frauen waren ähnlich wie in anderen Screeningprogrammen auf Gefäßerkrankungen überproportional häufig repräsentiert [3]. Unter den Teilnehmern befanden sich kaum Diabetiker zwischen 40 und 49 Jahren, die Mehrzahl der Teilnehmer erfüllte das Einschlusskriterium des Alters von mindestens 60 Jahren.

Die niedrige Prävalenz von Gefäßerkrankungen bzw. -veränderungen bei den Studienteilnehmern erscheint überraschend, da Ergebnisse des Dare-to-C.A.R.E.-Programms und anderer kombinierter Gefäßscreenings aus anderen Ländern eine deutlich höhere Prävalenz abklärungs- und interventionsbedürftiger Befunde aufwiesen [21, 26]. Ein Grund hierfür könnte das unterschiedliche spezifische Risikoprofil der gescreenten Populationen darstellen. So waren in der ursprünglichen Kohorte des Dare-to‑C.A.R.E.-Programms in den USA 59 % der Personen aktive oder ehemalige Raucher, 46,7 % litten an einer arteriellen Hypertonie, 49,4 % an einer Hyperlipidämie, 11,9 % der Teilnehmer hatten anamnestisch einen Myokardinfarkt erlitten. Dies stellt eine deutlich höhere Prävalenz kardiovaskulärer Risikofaktoren bzw. Vorerkrankungen als in der vorliegenden Studienpopulation dar. Die überproportional häufige Teilnahme von Frauen an unserem Screeningprogramm könnte ein weiterer Grund für die niedrige Prävalenz weiter abklärungs- und behandlungsbedürftiger Befunde sein. Da bei Frauen arteriosklerotische Veränderungen wie das AAA seltener und erst in höherem Alter auftreten, wird für sie in der Regel eine Teilnahme an von Gesundheitssystemen getragenen AAA-Screenings nicht empfohlen. Auch Studien zum Screening mit einem hohen Anteil an Frauen in der untersuchten Population sind selten [11, 13, 27]. Darüber hinaus könnte eine im Länder- und Systemvergleich unterschiedliche Bereitschaft von Menschen mit einem besonders hohen oder niedrigen Risiko für Gefäßerkrankungen, an Screeningprogrammen teilzunehmen, eine Rolle spielen [28]. Auch die Struktur der primären Gesundheitsversorgung differiert stark zwischen verschiedenen Systemen. In Deutschland ist die haus- und fachärztliche Versorgung im ambulanten Sektor sehr umfassend ausgeprägt, sodass bei vielen Personen ein informales Screening auf kardiovaskuläre Risikofaktoren und in gewissem Umfang auch bei asymptomatischen Personen auf das Vorliegen von Gefäßerkrankungen durchgeführt wird, die Diagnosestellung somit außerhalb eines institutionalisierten Screeningprogramms erfolgt. Es existieren auch innerhalb Deutschlands geografische Unterschiede u. a. in Bezug auf eine geringere Ärztedichte besonders in ländlichen Gebieten sowie im Vergleich der neuen und alten Bundesländer [4].

Andere nichtselektive Screeningprogramme aus den USA und europäischen Ländern zeigten hingegen Ergebnisse, die den unsrigen wesentlich ähnlicher waren. So wurden in Kalifornien in einem Zeitraum von 18 Monaten 1719 Personen auf das Vorliegen eines AAA, einer Karotisstenose und einer pAVK gescreent. Hiervon wiesen 0,3 % eine > 60 %ige Stenose, 1 % ein behandlungsbedürftiges AAA und 5,8 % einen ABI < 0,9 auf [3]. Die Prävalenz eines maximalen transversalen Aortendurchmessers > 3 cm bei erstmals zum Screening eingeladenen Männern in England und Schweden betrug 1,3–1,7 % und ist somit ebenfalls mit den Ergebnissen der vorliegenden Studie vergleichbar [11, 13, 27].

Eines der Ziele des Screeningprogramms stellte die Patientenbindung an die durchführende Einrichtung dar. Erhobene behandlungsbedürftige Befunde sollten mit den Hausärzten der Teilnehmer rückgekoppelt werden. Selbstverständlich hätte keine Verpflichtung bestanden, den Patienten dann dem Studienzentrum zur regulären Behandlung zuzuweisen. Es war aber davon auszugehen, dass dies für die meisten Patienten und Hausärzte die primäre Anlaufstelle ist. Letztendlich wurde jedoch in der gesamten Studienpopulation lediglich bei 2 Teilnehmern ein Befund (Stenose der A. carotis interna) festgestellt, bei dem eine Intervention zumindest in Erwägung zu ziehen war. Zusammenfassend muss konstatiert werden, dass das untersuchte Screeningprogramm in seiner vorliegenden Form im deutschen Gesundheitssystem keine Portalfunktion für die durchführende Klinik erfüllt. Inwiefern Menschen mit einer zu erwartenden höheren Prävalenz an Gefäßerkrankungen durch Infomaßnahmen erreicht und besser zur Wahrnehmung von Screeninguntersuchungen motiviert werden könnten, müssten weiterführende Studien klären.

Screeningprogramme werden häufig kontrovers beurteilt. Ihr Nutzen muss sich an patientenrelevanten Endpunkten wie beispielsweise der Reduktion der Gesamtmorbidität messen lassen. Sie sollten nicht ungerichtet in einer unselektionierten Population angewendet werden [15, 20, 29]. Die vorliegende Studie erlaubt keine Quantifizierung des potenziellen Nutzens des eingesetzten Gefäßscreeningprogramms. Vielmehr sollte in einem explorativen Design die Machbarkeit des Programms evaluiert werden und durch explorative Analysen Daten zu Charakteristika der Teilnehmer des Programms sowie erste Informationen zur Prävalenz der als Zielkriterien definierten Risikofaktoren und Gefäßerkrankungen gewonnen werden.

Der Nachweis einer Erkrankung im Rahmen eines Screenings kann zu psychischen Belastungen in Form von Krankheitsängsten und existenziellen Sorgen führen [11]. Dieser Aspekt konnte in unserer Studie durch die erhobenen Daten nicht näher betrachtet werden, muss aber bei Screeningprogrammen immer Beachtung finden und unter Umständen durch spezifische Aufklärungs- und Betreuungsmaßnahmen adressiert werden.

## Schlussfolgerung

Zusammenfassend zeigte diese Studie die Durchführbarkeit eines Screeningprogramms auf das Vorliegen einer Karotisstenose, einer pAVK und eines AAA bei Personen, die über spezifische Infomaßnahmen in Haus- und Facharztpraxen, im Krankenhaus und in konventionellen und sozialen Medien eingeladen wurden. Im Rahmen des Programms wurden kaum behandlungsbedürftige Gefäßpathologien nachgewiesen, sodass ein flächendeckender Einsatz in Deutschland in der vorliegenden Form auf Basis der verfügbaren Daten derzeit nicht empfohlen werden kann.
